# Dynamic Modeling of Vaccinating Behavior as a Function of Individual Beliefs

**DOI:** 10.1371/journal.pcbi.1000425

**Published:** 2009-07-10

**Authors:** Flávio Codeço Coelho, Claudia T. Codeço

**Affiliations:** 1 Theoretical Epidemiology Group, Instituto Gulbenkian de Ciência, Oeiras, Portugal; 2 Scientific Computing Program, Oswaldo Cruz Foundation, Rio de Janeiro, Rio de Janeiro, Brazil; Emory University, United States of America

## Abstract

Individual perception of vaccine safety is an important factor in determining a person's adherence to a vaccination program and its consequences for disease control. This perception, or belief, about the safety of a given vaccine is not a static parameter but a variable subject to environmental influence. To complicate matters, perception of risk (or safety) does not correspond to actual risk. In this paper we propose a way to include the dynamics of such beliefs into a realistic epidemiological model, yielding a more complete depiction of the mechanisms underlying the unraveling of vaccination campaigns. The methodology proposed is based on Bayesian inference and can be extended to model more complex belief systems associated with decision models. We found the method is able to produce behaviors which approximate what has been observed in real vaccine and disease scare situations. The framework presented comprises a set of useful tools for an adequate quantitative representation of a common yet complex public-health issue. These tools include representation of beliefs as Bayesian probabilities, usage of logarithmic pooling to combine probability distributions representing opinions, and usage of natural conjugate priors to efficiently compute the Bayesian posterior. This approach allowed a comprehensive treatment of the uncertainty regarding vaccination behavior in a realistic epidemiological model.

## Author Summary

A frequently made assumption in population models is that individuals make decisions in a standard way, which tends to be fixed and set according to the modeler's view on what is the most likely way individuals should behave. In this paper we acknowledge the importance of modeling behavioral changes (in the form of beliefs/opinions) as a dynamic variable in the model. We also propose a way of mathematically modeling dynamic belief updates which is based on the very well established concept of a belief as a probability distribution and its temporal evolution as a direct application of the Bayes theorem. We also propose the use of logarithmic pooling as an optimal way of combining different opinions which must be considered when making a decision. To argue for the relevance of this issue, we present a model of vaccinating behaviour with dynamic belief updates, modeled after real scenarios of vaccine and disease scare recorded in the recent literature.

## Introduction

Since early vaccination campaigns against smallpox, vaccination policies have been a matter of debate [1]: mass vaccination versus blocking strategies; compulsory versus voluntary, are some highly debated issues. Despite these early controversies - and consequent alternative policies implemented in different countries - high disease scare in the past has led to very high vaccine coverage and consequent successful eradication of smallpox, as well as very low incidence of measles, polio, tetanus, diphtheria, etc, resulting in over 98% mortality reduction by vaccine preventable diseases in developed countries [2].

In recent years, after complete or almost complete elimination of these diseases, the debate is shifting towards issues of vaccine safety. Increased perception of vaccine risks and lowered perception of disease risks has challenged previous willingness to vaccinate (fundamental for the success of any immunization program, either voluntary or compulsory) [3]. In this scenario, understanding and predicting individual's willingness to vaccinate is paramount for estimating vaccine coverage and compare strategies to achieve coverage goals.

Willingness to vaccinate is highly dependent on the perceived risk of acquiring a serious disease [4]. When (perceived) disease risk is low, however small risk of adverse events from the vaccine become relatively important and may lead to vaccine coverage lower than required to control transmission [4]. When (perceived) serious disease risk is too high, on the other hand, vaccine coverage may increase above that required to guarantee population protection [5]. We illustrate these behaviors with two examples:

### The MMR vaccine scare

In the UK, MMR vaccine uptake started to decline after a controversial study linking MMR vaccine to autism [6]. In a decade, vaccine coverage went well below the target herd immunity level of 95%. Despite the confidence of researchers and most health professionals on the vaccine safety, the confidence of the public was deeply affected. In an attempt to find ways to restore this confidence, several studies were carried out to identify factors associated with parent's unwillingness to vaccinate their children. They found that ‘Not receiving unbiased and adequate information from health professionals about vaccine safety’ and ‘media's adverse publicity’ were the most common reasons influencing uptake [7]. Other important factors were: ‘lack of belief in information from the government sources’; ‘fear of general practitioners promoting the vaccine for personal reasons’; and ‘media scare’. Note that during this period the risk of acquiring measles was very low due to previously high vaccination coverage.

### The Brazilian Yellow Fever disease scare

**Figure 1:**
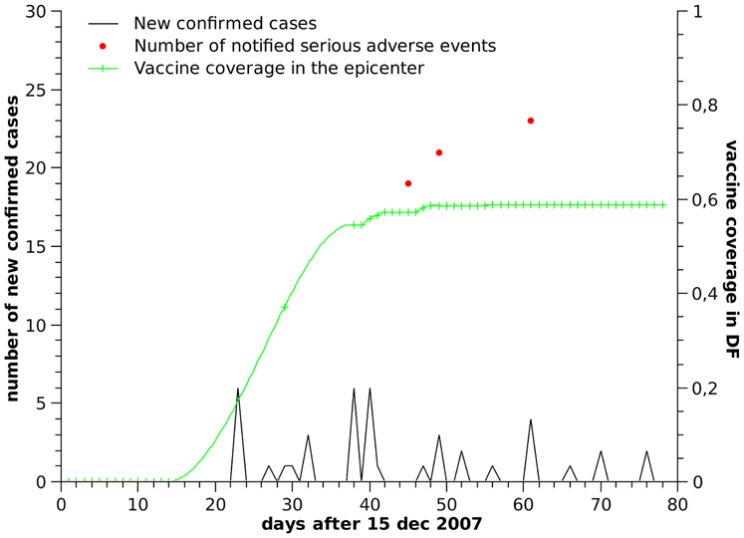
Yellow fever cases, vaccination coverage, and adverse vaccination events. Coverage data is from Brasilia, which was the epicenter of the outbreak.

Sylvatic yellow fever (SYF) is a zoonotic disease, endemic in the north and central regions of Brazil. Approximately 10% of infections with this flavivirus are severe and result in hemorrhagic fever, with case fatality of 50% [8]. Since the re-introduction of A. aegypti in Brazil (the urban vector of dengue and yellow fever), the potential reemergence of urban yellow fever is of concern [9]. In Brazil, it is estimated that approximately 95% of the population living in the yellow fever endemic regions have been vaccinated. In this area, small outbreaks occur periodically, especially during the rainy season, and larger ones are observed every 7 to 10 years [10], in response to increased viral activity within the environmental reservoir. In 2007, increased detection of dead monkeys in the endemic zone, led the government to implement vaccine campaigns targeting travellers to these areas and the small fraction of the resident population who were still not protected by the vaccine. The goal was to vaccinate 10–15% of the local population. Intense notification in the press regarding the death of monkeys near urban areas, and intense coverage of all subsequent suspected and confirmed human cases and death events led to an almost country-wide disease scare (Figure 1), incompatible with the real risks [5], which caused serious economic and health management problems, including waste of doses with already immunized people (60% of the population was vaccinated when only 10–15% would be sufficient), adverse events from over vaccination (individuals taking multiple doses to ‘guarantee’ protection), national vaccine shortage and international vaccine shortage, since Brazil stopped exporting YF vaccine to supply domestic vaccination rush (www.who.int/csr/don/2008_02_07/en/).

The importance of public perceptions and collective behavior for the outcome of immunization campaigns are starting to be acknowledged by theoreticians [9],[11],[12]. These factors have been examined in a game theoretical framework, where the influence of certain types of vaccinating behaviour on the stability and equilibria of epidemic models is analyzed.

In the present work, we propose a model for individual immunization behavior as an inference problem: Instead of working with fixed behaviors, we develop a dynamic model of belief update, which in turn determines individual behavior.

An individual's willingness to vaccinate is derived from his perception of disease risk and vaccine safety, which is updated in a Bayesian framework, according the epidemiological facts each individual is exposed to, in their daily life. We also explore the global effects of individual decisions on vaccination adherence at the population level.

In summary, we propose a framework to integrate dynamic modeling of learning (belief updating) with decision and population dynamics.

## Results

**Figure 2:**
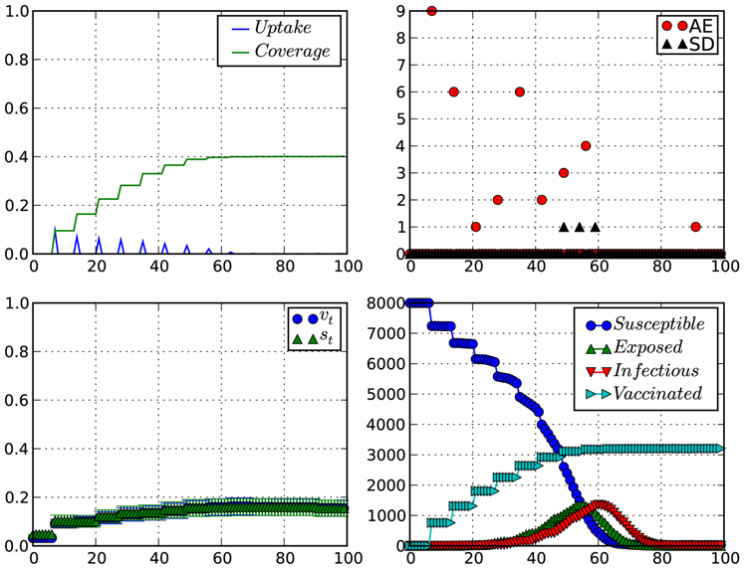
Impact of occurrence of adverse vaccination events at the beginning of a vaccination campaign. Top-left: vaccination coverage and vaccine uptake (×8000 doses);top-right: adverse vaccination and serious disease events; Bottom-left: willingness to vaccinate (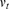) and perceived vaccine safety (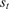); Bottom-right: Epidemiological time-series. Time is in weeks. 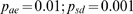.

**Figure 3:**
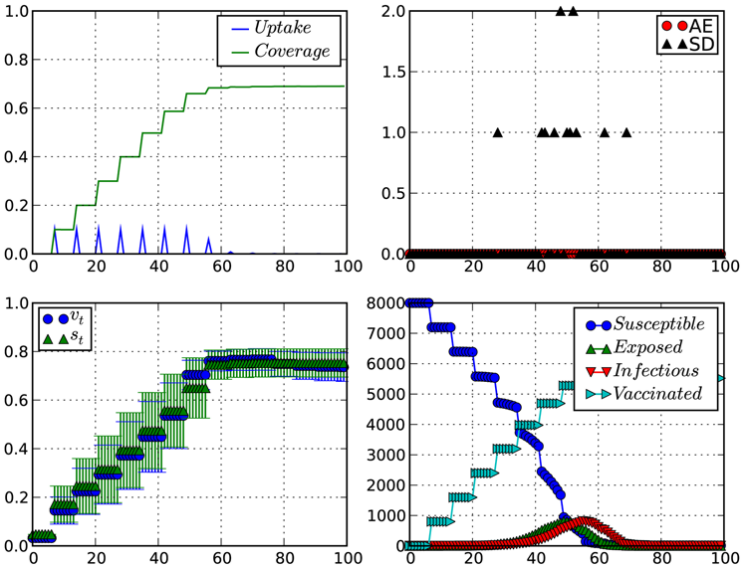
Impact of occurrence of serious disease events at the beginning of a vaccination campaign. Top-left: vaccination coverage and vaccine uptake (×8000 doses);top-right: adverse vaccination and serious disease events; Bottom-left: willingness to vaccinate (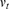) and perceived vaccine safety (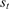); Bottom-right: Epidemiological time-series. Time is in weeks. 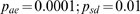.

We ran the model as described above for 100 days with parameters given by Table 1, under various scenarios to reveal the interplay of belief and action under the proposed model. Figures 2 and 3 show a summary output of the model dynamics under contrasting conditions. In Figure 2, we have VAE (Vaccine adverse events) preceding the occurrence of severe disease events. As expected, VAE become the strongest influence on 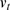, keeping 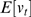 low with consequences to the attained vaccination coverage at the end of the simulation. We characterize this behavior as a ‘vaccine scare’ behavior.

**Table 1:** Parameter values used for the simulations.

Symbol	Meaning	Value
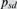	prob. of serious illness	[10^−6^,10^−2^]
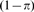	prob. of vaccine adverse effects	[10^−6^,10^−2^]
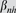	prob. of transmission/contact	0.2
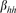	prob. of transmission/contact at home	0.3
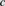	number of contacts per day	4
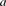	media amplification factor	[1,16]

In a different scenario, Figure 3, we observe the effect of severe disease events occurring in high frequency at the beginning of the epidemics. In this case, disease scare pushes willingness to vaccinate (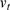) to high levels. This is very clear in Figure 3 where there is a cluster of serious disease cases around the 30th day of simulation. right after the occurrence of this cluster, we see 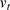 rise sharply above 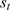, meaning that willingness to vaccinate (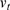) in this week was mainly driven by disease scare instead of considerations about vaccine safety(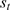). A similar effect can be observed in Figure 2, starting from day 45 or so. Only here the impact of a cluster of serious disease cases is diminished by the effects of VAEs, and the fact that there aren't many people left to make the decision of wether or not vaccinate.

**Figure 4:**
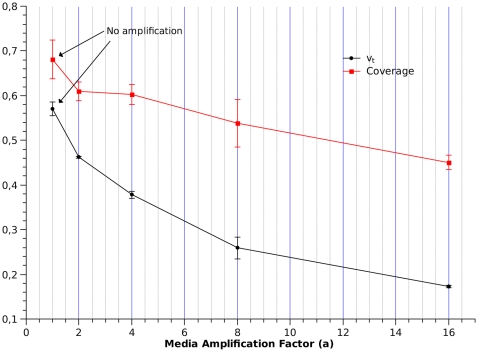
Effect of media amplification factor on vaccination coverage and willingness to vaccinate. Coverage and 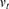 values are averages and standard deviations over the population values in the last week of simulation.

The impact of individual beliefs on vaccine coverage is highly dependent on the visibility of the rare VAE. Figure 4 shows the impact of the media amplification factor on 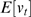 and vaccination coverage after ≈14 weeks, for a infectious disease with 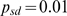 and 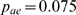. If no media amplification occurs, willingness to vaccinate and vaccine coverage are high, as severe disease events are common and severe adverse events are relatively rare. As vaccine adverse events are amplified by the media, individual's willingness to vaccinate at the end of the 14 weeks tend to decrease. Such belief change, however, has a low impact on the vaccine coverage. The explanation for this is that vaccine coverage is a cumulative measure and, when VAE appear, a relatively large fraction of the population had already been vaccinated. These results suggest that VAE should not strongly impact the outcome of an ongoing mass vaccination campaign, although it could affect the success of future campaigns.

**Figure 5:**
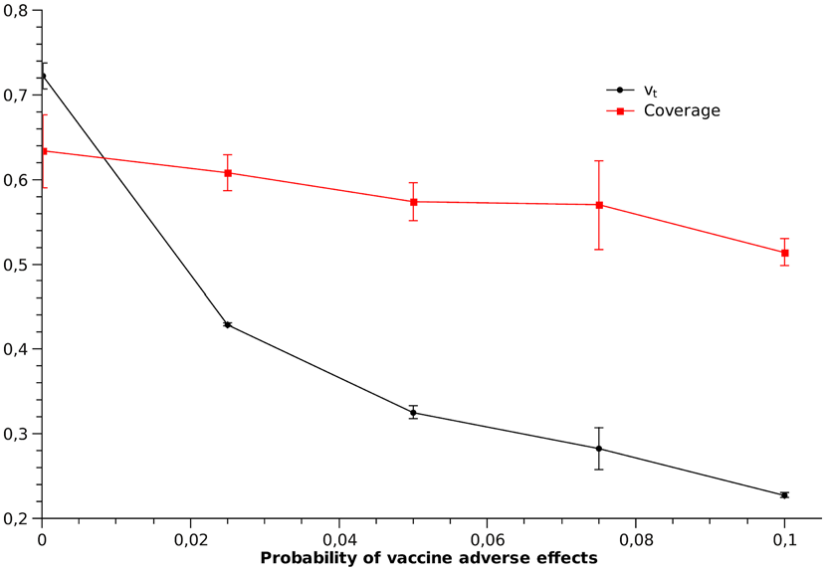
Vaccine coverage and willingness to vaccinate for vaccines with different levels of safety. Coverage and 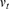 values are averages and standard deviations over the population values in the last week of simulation.

Fixing amplification at 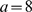 and 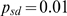, we investigated how 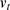 (at the end of the simulation) and vaccine coverage would be affected by increasing the rate of vaccine adverse events, 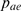 (Figure 5). As 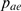 increases above 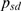, willingness to vaccinate drops quickly, while vaccine coverage diminishes but slightly.

## Discussion

In the present world of mass media channels and rapid and inexpensive communications, the spread of information, independent of its quality, is very effective, leading to considerable uncertainty and heterogeneity in public opinions. The yellow fever scare in Brazil demonstrated clearly the impact of public opinion on the outcome of a vaccination campaign, and the difficulty in dealing with scare events. For example, no official press release was taken at face value, as it was always colored by political issues [5]. In multiple occasions, people reported to the press that they would do the exact opposite of what was being recommended by public health authorities due to their mistrust of such authorities. This example shows us the complexity of modeling and predicting the success of disease containment strategies.

The goal of this work was to integrate into a unified dynamical modeling framework, the opinion and decision components that underlie the public response to mass vaccination campaigns, specially when vaccine or disease scares have a chance to occur. The proposed analytical framework, although not intentionally parameterized to match any specific real scenario, qualitatively captured the temporal dynamics of vaccine uptake in Brasilia (Figure 1), a clear case of disease scare (compare with simulation results, presented on Figure 2).

After conducting large scale studies on the acceptance of the Influenza vaccine, Chapman et al. [13] conclude that perceived side-effects and effectiveness of vaccination are important factors in people's decision to vaccinate. Our model suggests that, if the perception of disease risk is high, it leads to a higher initial willingness to vaccinate, while adverse events of vaccination, even when widely publicized by the media, tend to have less impact on vaccination coverage. VAE are more effective when happening at the beginning of vaccination campaigns, when they can sway the opinions of a larger audience. Although disease scare can counteract, to a certain extent the undesired effects of VAE, public health officials must also be aware of the risks involved in overusing disease risk information, in vaccination campaign advertisements since this can lead to a rush towards immunization as seen in the 2008 Yellow Fever scare in Brazil.

Vaccinating behavior dynamics has been modelled in different ways in the recent literature, from behaviors that aim to maximize self-interest [12] to imitation behaviors [14]. In this paper we modeled these perceptions dynamically, and showed its relevance to decision-making dynamics and the consequences to the underlying epidemiological system and efficacy of vaccination campaigns. We highlight two aspects of our modeling approach that we think provide important contributions to the field.

First, the process through which people update beliefs which will direct their decisions, was modeled using a Bayesian framework. We trust this approach to be the most natural one as the Bayesian definition of probability is based on the concept of belief and Bayesian inference methodology was developed as a representation human learning behavior [15]. The learning process is achieved through an iterative incorporation of newly available information, which naturally fit into the standard Bayesian scheme. Among the advantages of this approach is its ability to handle the entire probability distributions of the parameters of interest instead of operating on their expected values which would be the cased in a classical frequentist framework. This is especially important where highly asymmetrical distributions are expected. The resulting set of probability distributions, provides more complete model-based hypotheses to be tested against data. The inferential framework has an added benefit of simplicity and computational efficiency due the use of conjugate priors, which gives us a closed-form expression for the Bayesian posterior without the need of complex posterior sampling algorithms such as MCMC.

The second contribution is the articulation between the belief and decision models through logarithmic pooling. Logarithmic pooling has been applied in many fields [16],[17] to derive consensus from multiple expert opinions described as probability distributions. Genest et al. [15], argue that Logarithmic pooling is the best way to combine probability distributions due to its property of “external Bayesianity”. This means that finding the consensus among distributions commutes with revising distributions using the Bayes formula, with the consequence that the results of this procedure can be interpreted as a single Bayesian probability update. Here, we apply logarithmic pooling to integrate the multiple sources of information (equation (1)) which go into the decision of whether or not to vaccinate. In this context, the property of external bayesianity, is important since it allows the operations of pooling and Bayesian update (of 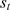, equation (2)) to be combined in any order, depending only on the availability of data.

This framework can be easily used as a base to compose more complex models. Extended models might include multiple beliefs as a joint probability distribution, more layers of decision or multiple, independently evolving belief systems.

The contact strucure of the model was intentionally kept as simple as possible, since the goal of the model was to focus on the belief dynamics. Therefore, a reasonably simple epidemiological model, with a simple spatial structure (local and global spaces) was constructed to drive the belief dynamics without adding potentially confounding extra dynamics.

In this work we have played with various probability levels of VAEs and SDs in an attempt to cover the most common and likely more interesting portions of parameter space. However, to model specific scenarios, data regarding the actual probabilities of VAEs and SDs are a pre-requisite. Also important are data regarding the perception of vaccine safety and efficacy [18], obtainable through opinion surveys which could also include questions about factors driving changes in vaccination behavior. We therefore suggest that questions regarding these variables should be included in future surveys concerning vaccine-preventable diseases. This would improve our ability to predict of the outcome of vaccination campaigns.

## Materials and Methods

We set the vaccination decision problem in the context of a population experiencing a vaccine preventable disease outbreak which leads to a mass vaccination campaign. Individuals receive information regarding vaccine and disease events from local and global sources. We assume that 'good' events (prompt recovery from infection or safe vaccine events) are visible locally only while severe cases of disease or potentially adverse events from the vaccine enjoy global visibility due to the natural preference of media channels for scary stories. In order to integrate behavioral and epidemiological dynamics, an individual based model was developed. Individual's behavior regarding vaccination is represented in a belief-decision model which describes the dynamics of belief updates in response to epidemiological events and the decision making based on the person's current beliefs. The epidemiological model determines the disease dynamics in a population with hierarchical contact structure, representing a large urban setting.

### Belief model

The belief model describes the temporal evolution of each individual's willingness to vaccinate, 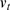, in response to his evaluation of vaccine safety and disease risk. To account for the uncertainties regarding vaccinating behavior, 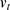 is modeled as a random variable, whose distribution is updated weekly as the individual observes new events. The update process is based on logarithmically pooling 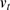 with other random variables as described below. Logarithmic pooling is a standard way of combining probability distribution representing opinions, to form a consensus [15].

The belief update model takes the form:
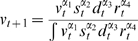
(1)


where 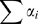 must equal one as 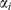 act as weights of the pooling operation. We attributed equal weights to 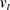 and 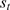 (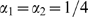), with remaining 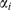 taking values according to the following conditions:
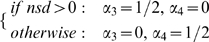
where 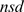 is the number of serious disease cases witnessed by the individual, and 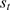 and 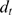 are random variables describing individual's belief regarding vaccine safety and disease risk, respectively. The values for 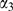 and 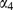 are set to 1/2 since either 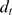 or 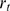 are to be pooled against the combination of 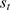 and 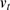: 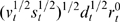. This choice of weights corresponds to the most unassuming scenario regarding the relative importance of each information source, different weights may be chosen for different scenarios. Every individual starts off with a very low expected value for the Beta-distributed 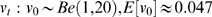.

The last term in (1), 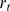, is a reduction force which causes 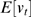 to move towards the minimum value of 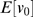. This term is important since without it, the psychological effects of witnessing serious disease events would continue to influence the individual's decisions for and indetermined period of time. Thus, 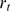 allows us to include the memory of such events in the model. By setting 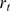 appropriately, we can model events that leave no memory as well as ones that are retained indefinetly.

#### Perceived vaccine safety (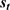)

Regularly, during a mass vaccination campaign, individuals will try to infer the value of vaccinating based on available information regarding vaccine events. During a campaign, the number of safe vaccine events, 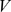 can naturally be modeled as a binomially distributed variable, with parameters 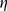 and 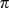 standing for the number of doses given and the probability of safe event, respectively [19]. However, since data available to individuals is biased and incomplete, the observed variable 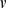 that feeds each individual's inferential process is a Binomial(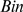) governed by 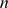 and 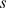, the perceived number of doses and perceived probability of a safe vaccine event, respectively:
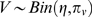


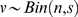



Note that 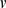 is not the true number of safe vaccine doses applied in the population, but represents a subset of these events which the individual is aware of. This means that the perceived safety of the vaccine will always be a biased estimate, and will vary from individual to individual generating variation in the population belief distribution.

**Figure 6:**
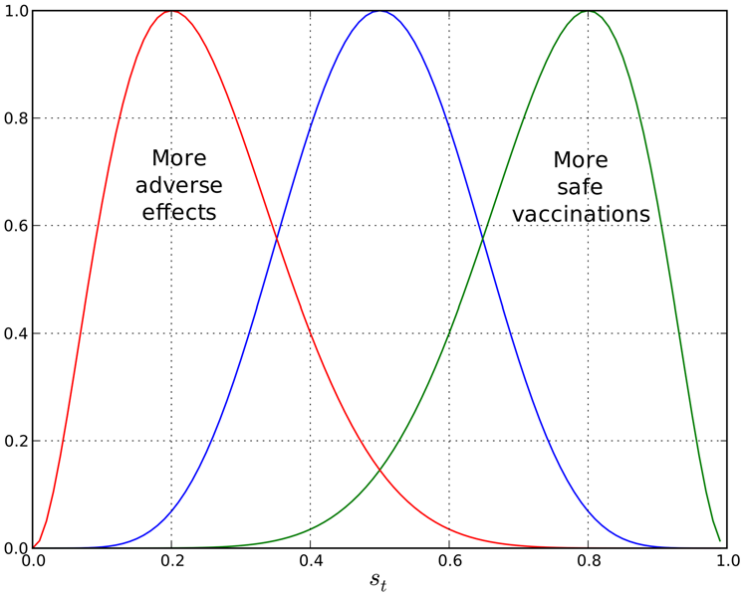
Updating the perceived probability of a safe vaccination. The expected value of 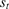 will change towards one with more safe vaccines witnessed and in the opposite direction with the accumulation of vaccine adverse effects (VAE) events. Y-axis are arbitrary units.

Each individual will make inference of 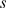 based on 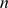 and 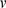, This is modelled as a iterative Bayesian inference. Let the prior distribution for 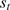 be a Beta distribution, 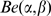, which is the natural conjugate for a binomial process. The posterior distribution 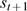 is then given by:
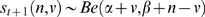
(2)The posterior 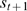 is used in the subsequent iteration cycle as the prior (Figure 6).

To better emulate the biased availability of good versus bad news in real populations, we assume that vaccine adverse events are visible globally, while safe events are visible only within their neighborhoods. To include the effects of an exaggerated media coverage of vaccine adverse events, we considered scenarios where the the observed number of adverse events 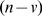 is amplified by a constant 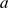 in equation (2), which then becomes 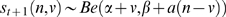. We call this factor (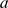) the “media-amplification factor'' , which varies from 1 (no amplification) to 16 in our simulations. The chosen range for this factor, has no bearings in any real data, but instead was selected to be just enough to demonstrate the sensitivity of the model to such an effect. If real data is available, it may still require transforming to match this intensity range in order to be properly incorporated into the model.

#### Perceived disease risk (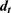)

In this model, we try to emulate a scary disease, that is, a disease severe enough that a few cases will lead to a high willingness to get a vaccine shot.

**Figure 7:**
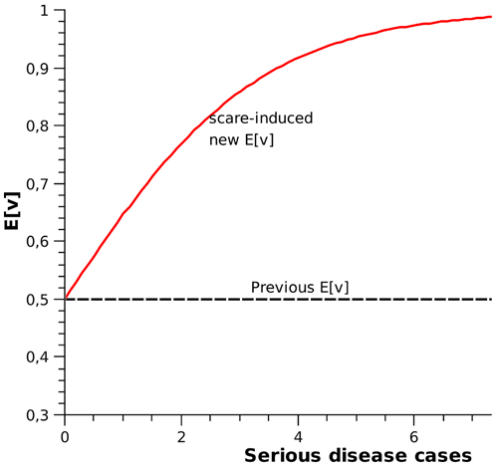
Mean scare as a function of Serious disease cases witnessed.

Disease scare is defined as an increase in the individual's 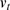, upon witnessing disease cases with serious consequences. It must be noted, however, that this probability refers to the decision of getting a vaccine, since effectively getting vaccinated will also depend on the availability of the vaccine. This effect enters 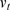 update equation (1) as the variable 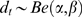, where:
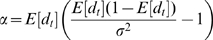
(3)

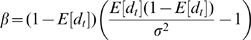


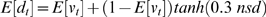
and 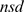 is the number of serious disease cases witnessed. To put it plainly, equation-set (3) shows how one can obtain the parameters of a Beta distribution from its expected value, and demonstrates how the expected value of 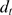 is calculated from serious disease cases. Figure 7 shows how 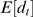 varies with the number of serious disease cases witnessed. Serious disease cases are visible globally. Here, 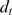 has a fixed variance 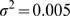. The pooling between 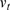 and 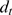 is done as in (1). In equation (1) 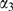 can be modified to make the disease more or less scary to individuals.

#### Reduction (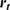)

The reduction term is a slow but continuous change of the mean willingness to vaccinate, 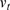 towards its initial distribution 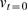. This will happen only in the absence of perceived serious disease cases. The reduction term enter 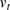 update cycle as the variable 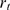 in (1) and has a Beta distribution with mean given by:
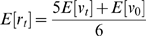
and parameters derived from 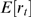 in the same way as in (3). The reduction term is calculated as a weighted average between the current probability to vaccinate, 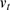, and the initial probability to vaccinate, 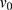, at the beginning of the simulation. the weights in this average can be modified to change the magnitude of the reduction term (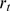) in equation (1).

#### Decision

**Figure 8:**
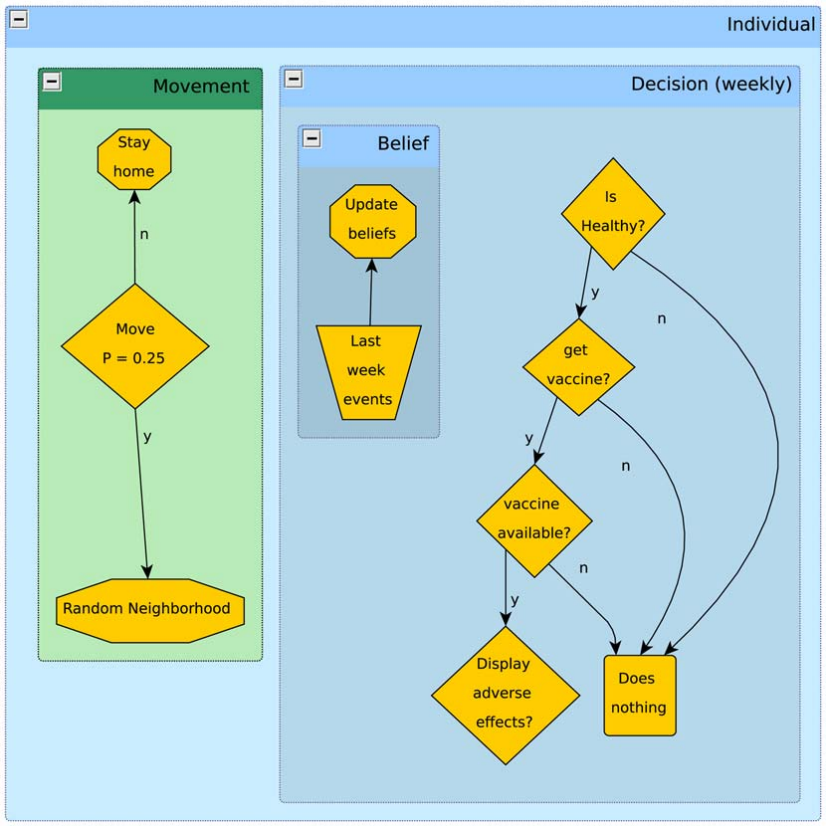
Daily and weekly events that determine individual activity. Movement decisions take place on a daily basis.

Once a week, during the simulation, susceptible and exposed individuals decide whether to go vaccinate with a probability sampled from 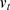, updated according to equations (1) and (2). This update is based on evidence collected during the past seven days (Figure 8).

Only non-infectious individuals make the decision to whether or not they should go vaccinate. We consider that exposed individuals do not know they have been infected, so they also may seek vaccination. This is important because there is a limited amount of vaccine doses available per week and exposed individuals will compete with susceptibles for them. Only susceptibles are successfully immunized by the vaccine.

### Population model

**Figure 9:**
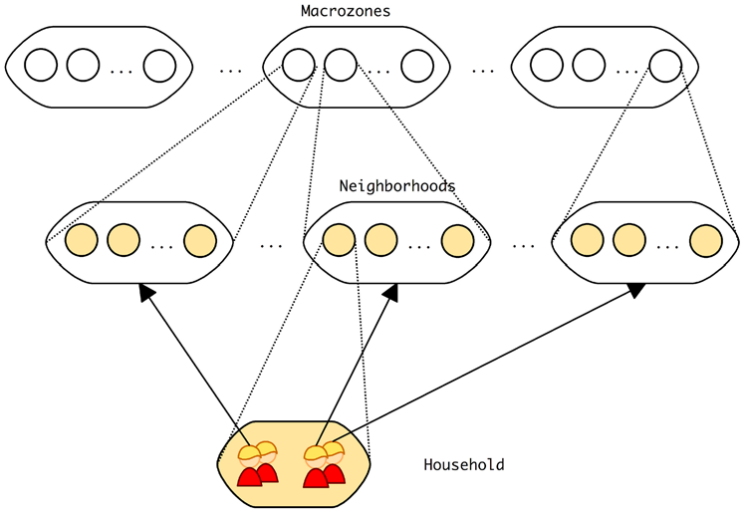
Design of the hierarchical population model. Arrows show the possible patterns of daily movement of individuals.

We model disease spread in a hypothetical city represented by a multilevel metapopulation individual-based model where individuals belong to groups that in turn belong to groups of groups, and so on (Figure 9), forming a hierarchy of scales [20]. In this hypothetical city, individuals live in households with exactly 4 members each; neighborhoods are composed by 100 households and sets of 10 neighborhoods form the city's zones. During the simulation, individuals commute between home and a randomly chosen neighborhood anywhere in the population graph. Each individual has a probability 0.25 of leaving home daily.

This same hierarchical structure is used to define local and global events. Locally visible events can only be witnessed by people living in the same neighborhood while globally visible events are visible to the entire population regardless of place of residence.

### Epidemiological model

The epidemiological model describes a population being invaded by a new pathogen. This pathogen causes an acute infection, lasting 11 days (incubation period of 6 days and an infectious period of 5 days). Once in the infectious period, individuals have a fixed probability, 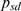 of becoming seriously ill. After recovery, individuals become fully immune. The proportion of the population in each immunological state at time 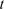 is labeled as 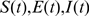 and 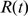, which stands for susceptibles, exposed, infectious and recovered states.

At the same time the disease is introduced in the population, a vaccination campaign is started, making available 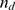 doses per week to the entire population, meaning that individuals may have to compete for a dose if many decide to vaccinate at the same time.

Once an individual is vaccinated, if he/she has not been exposed yet, he/she moves directly to the recovered class, with full immunity (thus, a perfect vaccine is assumed). If the individual is in the incubation period of the disease, disease progression is unaffected by vaccination. Vaccination carries with it a fixed chance 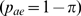 of causing adverse effects.

Transmission dynamics is modelled as follows: at each discrete time step, 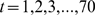, each individual contacts others in two groups: in his residence and in the public space. The probability of getting infected at home is given by 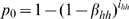 where 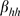 is the probability of transmission per household contact and 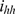 is the number of infected members in the house. In the public space, that is, in the neighborhood chosen as destination for the daily commutations, each infected person contacts 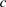 persons at random, and if the contact is with a susceptible, infection is transmitted with probability 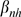.
